# Book Review

**Published:** 1923-01

**Authors:** 


### Reviews of Books.

**China and Modern Medicine.** By HAROLD BALME, F.R.C.S. Pp. 224. London: United Council for Missionary Education. 1921. Price 3s. 6d.—The progress of medical science in China is a subject of great interest from many points of view. Dr. Balme has given here, in outline, the history of a movement which will have a tremendous effect on the history of Asia and indeed the whole world. His conception of our duty to propagate the science of medicine in a nation civilised but practically devoid of any scientific knowledge cannot fail to impress his readers. Students of missionary enterprise will find in this book inspiration and abundant food for thought. To find medicine given its true place amongst the forces that exalt a nation is refreshing, and will appeal to those of utilitarian habit of thought. Suffering somewhat no doubt from the circumstances of its making, some rearrangement of matter would give a clearer idea of what is being done and what remains to be done in China, and of the author's conception of the best methods of laying the foundations of western medicine in the heart of the East. Those who have not lived there, and even some of those who have, have no idea of the tremendous difference there is between different parts of China in the degree in which western civilisation has penetrated. This is a vital point to grasp in trying to appreciate the present position of China in the family of nations, and much enlightenment herein may be had by those who read Dr. Balme's book carefully. Truly educative, this little book would be well worth a place in any library, even if it did nothing but show that the missionary has many more problems to exercise his brains than the man who stays at home.

**Tumours : Innocent and Malignant.** By Sir John Bland-Sutton, LL.D., F.R.C.S. Seventh Edition. Pp. x., 806. London: Cassell and Company, Ltd. 1922. Price 30s.—We are fortunate in having a new edition of this classical book, after an interval of five years from the last. At each new edition some additional information is added bearing on tumour growth, and to the present edition are also added twenty new and very excellent illustrations. Perhaps the most striking new statement in this edition is with regard to the supposed relationship of several forms of tumour growth to internal secretion. It has, of course, for some time been recognised that overgrowth of various tissues of the body may be due to an abnormal internal secretion of the pituitary and thyroid glands, but it is now believed that actual tumour growth may be also influenced in this way. Sir J. Bland-Sutton says there is reason for suspecting that multiple exostoses are the result of disturbance of the function of the glands of internal secretion—probably the thyroid, but he does not give us these reasons. He also says multiple fibro-neuromata and chondromata also probably depend on some disturbance of the endocrine glands, and that glandular disturbance of the hypophysis may cause multiple uterine fibroids. So that he considers multiple exostosis, chondromata and fibro-neuromata, and uterine fibroids may all be due to some abnormal conditions of internal secretion.

We are interested to notice that in the description of myeloma the author also describes the condition which has led to so many errors of diagnosis—errors which in a few cases have cost the patient the loss of a limb—innocent cysts of bone, and gives an illustration of a pathological specimen of one of these cysts from the College of Surgeons' Museum. He seems to consider that such a cyst may in some cases be really the remains of a myeloma. Of course, a myeloma may contain degeneration cysts, but it seems to us that it would be almost impossible to prove that an innocent cyst of bone started as a myeloma unless there were traces of the solid growth also present in a typical form, and not merely the presence of some giant cells in the wall of the cyst, where they are frequently found. Although, of course, on a strictly pathological basis a distended gall-bladder and a hydronephrotic kidney are as much retention cysts as a distended sebaceous gland duct, yet we regret that such conditions should still be described as such, and therefore included in this book on tumours. It is rather surprising that the "mixed tumour" of the testicle should not be described as an embryoma, which the latest investigations seem to indicate is its true nature. A very striking change in this new edition is that endotheliomata are no longer described. In the 5th edition such a tumour was described as occurring in eight different localities. This edition was published at the time when it was the fashion amongst pathologists to label any tumour of unusual microscopic structure an endothelioma. It has always seemed to us that it would have been better to have limited the scope of this work to the pathology of tumours, and not to have included their "clinical characters and appropriate treatment" in any detail. The clinical aspect of cancer of the stomach, colon, and rectum, and the advantages and risks of various forms of operation, have always seemed to us rather out of place in the book. It would have been better to omit it, for as we might expect it is not dealt with in really a thorough manner, as it is in the larger works dealing with abdominal surgery. But this new edition of Sir J. Bland-Sutton's book makes us realise afresh that the work is a large storehouse of knowledge with regard to the character of various tumours, and their relationship to each other and to allied morbid conditions; and its great originality, and the tendency of the author to dismiss as unproved various theories inadequately supported by facts, strikes us anew in this last edition.

**The Venereal Clinic.** By Several Writers. Edited by Ernest R. T. Clarkson, M.A., M.R.C.S. Pp. xvi., 477. London: John Bale, Son & Danielsson Ltd. 1922. Price 25s.

net.—This book serves to supply a deficiency in the literature of Venereal Disease in a most effective manner. It gathers up the essential points of all kinds, and places in the hands of all who are called upon to treat such cases the details which are required to make a satisfactory diagnosis by the most recent methods, as well as how to carry out treatment by the most up-to-date technique. It is not intended to be a comprehensive and exhaustive monograph, but as a " manual for the general practitioner and student attending Venereal Clinics." A short description is given of the diseases encountered and the essential points in diagnosis, the reader being carried along as with a case before him. In this way all the complications are considered and dealt with. The urethroscope is specially described, while bacteriology and laboratory methods are considered. The sociological and administrative aspects of the subject are given due place and medico-legal matters are not omitted. The book is well illustrated and printed, and can confidently be recommended to all students and persons interested in this section of medical practice.

**Anæsthetics in Practice and Theory.** By J. Blomfield, C.B.E., M.D. Pp. xii., 424. London: William Heinemann (Medical Books) Ltd. 1922. Price 25s. net.—Owing to his large experience and his philosophical turn of mind, Dr. Blomfield has succeeded in producing a work of exceptional value and attractiveness; in short, the book presents a very readable picture of the speciality, and blends old and new material without loss of perspective and with a remarkable absence of bias. The chapter on chemistry deals fully only with such bodies as are in common use, including Ethanesal, and explains that many of the substances with which Snow and others have experimented are not true anæsthetics as judged by the modern therapeutic standard. In discussing the physiological action of the various agents the author relates much of the experimental work published at home and abroad, but is careful not to commit himself to any opinion, except, perhaps, to favour the theory that anæsthesia depends upon an alteration in the nerve cells, but he does not attempt to show how this view can be reconciled with the fact that vegetable organisms can be anæsthetised. The section on administration is full of practical information, and will be appreciated, as helpful, by practitioners and students alike. The rather vexed question of when to use and when to avoid preliminary narcotic drugs is given careful consideration, and evokes a warning as to the danger of abolishing or unduly subduing the laryngeal reflex in the subjects of mouth and tongue operations. Local, spinal and sacral analgesia are dealt with at some length with clear descriptions of their respective techniques. In discussing the fatalities of anæsthesia the author lays stress on the truth of the " dictum " of the Anæsthetics Committee of the British Medical Association, which regards the anæsthetist's experience as the most important factor concerned, and he makes the significant comment in the following sentence : " Many a death which seems sudden to the administrator has that appearance only because he has not perceived early enough the indications of approaching danger." We feel confident that this book will quickly become very popular.—A.L.F.

**The Dictionary of Practical Medicine.** Edited by SIR MALCOLM MORRIS, K.C.V.O., FREDERICK LANGMEAD, M.D., F.R.C.P. Lond., and GORDON HOLMES, C.M.G., C.B.E., M.D. Dublin, F.R.C.P. Lond. London : Cassell & Co. Ltd. 1921. Three Volumes. Price £5 5s. od.—These books are written for the general practitioner, and are essentially practical in character, the chief aim being to assist him in the recognition and treatment of disease. Therefore each article lays more stress on symptomatology, diagnosis, prognosis and treatment than on etiology and pathology, though the two latter are not unduly ignored. They are copiously illustrated by plates, many of which are coloured, and also by other figures. The articles are arranged in alphabetical order, making it a very ready book for reference. The individual articles, written by over a hundred different contributors, are well up to date, and not only include general medicine but also such subjects as Immunity, Anæsthetics, Clinical Bacteriology and Pathology, the modern developments of Chemical Pathology, the Examination for Life Assurance, Forensic Medicine, Dermatology and Gynæcology. It is a work which we can heartily recommend to any busy practitioner.

**Functional Nervous Disorders.** By DONALD E. CORE, M.D., M.R.C.P. Pp. xvii., 371. Bristol: John Wright & Sons Ltd. 1922. Price 25s. net.—This book deals with classification and treatment. With regard to the former, the author adopts a classification based on evolutionary data, dividing functional disorders into regressive and progressive varieties. In the regressive group he places hysteria, which is further subdivided into primary, secondary and tertiary forms. In the progressive are placed, firstly, the sympathetic functional nervous disorders, namely the instinct-distortion neurosis, which are termed dysthymias, (*a*) confusional, or centrifugal, (*b*) introspective or centripetal, and, secondly, symptoms arising in the atmosphere of dread, comprising (*a*) the ordinary form of mnemoneurosis and (*b*) the obsessive form of mnemoneurosis. The first part of the book is devoted to general considerations, which include the behaviour of man compared with that in the lower animals. This is an important section. Subsequent parts consider in detail hysteria, the sympathetic nervous disorders, dreams, diagnosis, prognosis and treatment. There is a great deal that is original in this work, and much careful thought has been devoted to it. It is, however, too verbose in form, and many of the sentences are difficult to follow.

**A Glimpse into the History of the Surgery of the Brain.** The Thomas Vicary Lecture. By Sir Charles A. Ballance, K.C.M.G., C.B., M.V.O., M.S. Lond. Pp. 110. London: Macmillan and Co. Ltd. 1922. Price 10s. 6d. net.—The first half of this work is a brief survey of the general history of surgery, with occasional reference to the history of the surgery of the brain, doubtless because little was known of the latter subject apart from the very ancient practice of trephining and the Hippocratic methods of dealing with injuries of the head. In this connection, however, it might have been worth while to mention the fact that the Hippocratic school did not trephine for depressed fractures, and that the use of the trephine for such fractures of the skull was recommended by Avenzohar (A.D. 1113–1162), the teacher of the celebrated Averroës. The latter half of the book deals more consistently with the title of the lecture, for brain surgery, like general surgery, was based on sounder principles with the revival of anatomical knowledge in the Middle Ages. The work of Ferrier on localisation and the operative application of physiological and anatomical facts by such men as Horsley and Macewen marked a new epoch in the advance of brain surgery, and the author rightly claims that "during every decade since 1881 . . . the surgeon's art in cases of intracranial disease continues to triumph along many lines of advance." As a whole the book gives a clear but brief account of what is known of the history of the surgery of the brain from the earliest times to the present day. There are thirty illustrations, many of them being portraits of well-known pioneers of surgery.

**Ashby and Wright's Diseases of Children, Medical and Surgical.** Sixth Edition. Pp. xxvii., 796. London: Oxford Medical Publications. 1922. Price 42s. net.—This is the sixth edition of a well-known book written for senior students and junior medical practitioners. The original authors are both deceased, and the present book is a revision by Dr. H. T. Ashby, a son of one of them, in conjunction with Mr. Charles Roberts, F.R.C.S. The volume is a systematic text-book on pediatrics, and is concerned with diagnosis and treatment, the subject-matter being illustrated by a number of excellent plates. One of the earlier chapters deals with a most important and interesting branch of the subject, namely infant feeding, and abounds with much excellent advice and common sense; for example, under a description of the calorimetric method of feeding the authors state: "These calculations have been of value in giving a general idea of the quantity of food a baby needs, but in a calorimeter a baby does not behave like a baby in the nursery." Under this section the authors lay stress on the importance of regularly weighing infants. In a later chapter the writers are old-fashioned and wise enough to believe that there may actually be disorders of dentition, and furthermore include formulæ for medicines to treat such disorders. Certain other chapters are worthy of special commendation, notably that on Rickets; there is always a tendency either to exaggerate the importance of new discoveries or to ignore them, and it is gratifying to see that hygienic and climatic influence and environment are here considered to play at least as important a part as the accessory food factors in the etiology of this disease. The section on Diphtheria is also specially well written, and the important question of immunisation after the application of the Schick test is discussed. There is little need for serious criticism in any particular direction, but brief reference may be made to a few rather important points. Under "Delayed Chloroform Poisoning" and "Cyclic Vomiting" the word "acidosis" is used, and although the presence of acetone in the urine in these cases is referred to, no reference is made to the differences between "acidosis" and "Ketosis." The subject of "Tetany" and other spasmophilic conditions is worthy of fuller treatment, and, incidentally, no reference is made to the connection between tetany and the parathyroid glands. We also think that the use of the word "Eczema" to describe a clinical entity is hardly desirable. The book concludes with a helpful chapter on Radiography in Children by Dr. W. J. S. Bythell, a chapter on the teeth by Mr. B. J. Rodway, and appendices on methods, preparations, and formulæ. The authors are to be congratulated on their lucid method of presentation, the publishers on the clearness of the type, and both on the general excellence of the work as a whole.

**What to do in Cases of Poisoning.** By William Murrell, M.D., F.R.C.P. Twelfth Edition. Revised by P. Hamill, M.D., D.Sc., F.R.C.P. Pp. 273. London: H. K. Lewis & Co. Ltd. 1921. Price 4s. 6d. net.—In forty years Murrell's little pocket-book has established itself as a universal favourite. Each edition appears to be sold out in about three years. Every doctor in the country knows the book. It only remains for us to announce the issue of an up-to-date new edition ably edited by Dr. P. Hamill.

**Analyses and Energy Values of Foods.** By R. H. A. Plimmer, D.Sc. Pp. 255. London: His Majesty's Stationery Office. 1921. Price 6s. net.—The necessity for rationing food during the War directed public attention to the nutritive and energy values of the foodstuffs consumed. The Army Medical Authorities directed Dr. R. H. A. Plimmer (then a captain attached to the Directorate of Hygiene in the War Office) to draw up an exhaustive analytical report dealing with the subject. Dr. Plimmer's careful work will give this report a value far outlasting the War. It will probably be the standard reference book for the British Empire. For determining diet scales for institutions or for planning special diets for patients these tables are invaluable. The cost is very small, but the paper and type are excellent. Great credit is due to the War Office for authorising and to Dr. Plimmer for compiling these tables.

**Blood Transfusion.** By Geoffrey Keynes, M.A., M.D. Pp. vii., 166. London: Oxford Medical Publications. 1922. Price 8s. 6d. net.—This is a very interesting little book. It gives a clear account of the indications for blood transfusion and the best methods. These have often been given before, but the present volume is convenient. In addition, there is an interesting account of the history of the operation, and some valuable information about the national distribution of the four blood groups and the way in which they fit into Mendel's Law. Amongst Westerns Group II. is common; amongst Orientals Group III. Group I. means that the individual carries the factors that produce both the Group II. and the Group III. reaction; Group IV. means that both these are absent. It is well known that members of a family may belong to different blood groups, and the inheritance follows Mendel's Law. Thus if both parents are Group IV. the child cannot be anything but Group IV., and if the child belongs to Group I. it is strong evidence of illegitimacy; so too if the parents are both Group II. but the child is Group I. or III. The very important point is made, which I have observed myself, that a first transfusion for pernicious anæmia may be very successful, but a second transfusion long afterwards *from the same donor* may give rise to hæmoglobinuria, apparently from anaphylaxis. It is erroneously stated on page 121 that O. H. Robertson introduced the citrate method into the British Army in 1917. I am sure my friend Robertson would be the first to point out that we had been using the technique at a Casualty Clearing Station for months before we had the pleasure of welcoming him amongst us, and were already using the bottle method, which he slightly modified.—A.R.S.

